# Working with technical purity: simulation of red tattoo pigment metabolism by online-liquid chromatography-electrochemistry-mass spectrometry

**DOI:** 10.1007/s00216-024-05709-8

**Published:** 2025-01-04

**Authors:** Carina Wolf, Franziska Krall, Valentin Göldner, Uwe Karst

**Affiliations:** 1 Institute of Inorganic and Analytical Chemistry, University of Münster, Münster, Germany; 2 Department of Environmental Geosciences, Centre for Microbiology and Environmental Systems Science, University of Vienna, Vienna, Austria

**Keywords:** Tattoo pigments, Azo pigments, Metabolism simulation, Electrochemistry, Mass spectrometry

## Abstract

Tattooing is a popular form of body art that has evolved from ancient times into being part of modern society. The understanding of biotransformation processes of coloring tattoo pigments in human skin is limited although skin reactions to tattoos with unknown culprits occur. Electrochemistry coupled to mass spectrometry (EC-MS) has widely been used as a tool for a purely instrumental approach to simulating the enzymatic biotransformation of xenobiotics. For the application to pigments, however, clean standards are lacking. Hence, metabolic studies in general as well as simulation approaches like direct EC-MS are hindered because transformation products cannot be assigned unequivocally to the pigment or its impurities. This can be addressed by online separation prior to electrochemical transformation. An online-liquid chromatography (LC)-EC-MS approach was developed to simulate the metabolism of Pigment Red 5 (PR 5). The electrochemical cell was coupled to the chromatographic column and was operated in oxidative and reductive mode. Distinct retention times allowed the electrochemical transformation products to be linked to their parent compound. Oxidative and reductive azo cleavage of PR 5 were observed. Two different PR 5 formulations were investigated, yielding the same transformation products and showcasing the applicability to individual PR 5 samples with different impurity profiles. The presented LC-EC-MS approach overcomes the lack of clean standards that hampers the metabolic studies of tattoo pigments in general and using direct EC-MS without prior chromatographic separation. Applying the purely instrumental online separation approach, first ideas on possible transformation routes of pigments frequently identified in adverse skin reactions are provided.

**Graphical Abstract:**

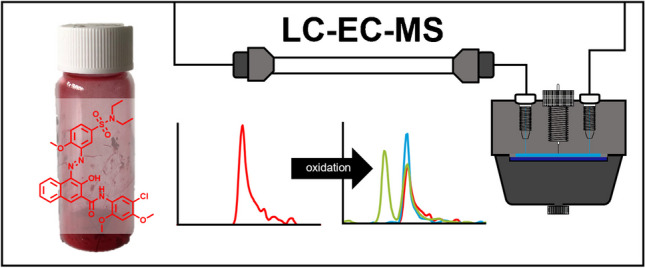

**Supplementary Information:**

The online version contains supplementary material available at 10.1007/s00216-024-05709-8.

## Introduction

In recent years, the body art tattooing has become increasingly popular throughout society. The main components of used tattoo inks are colorful pigments, providing the longevity of a tattoo due to their low solubility [1, 2]. In rare cases, tattooing can lead to skin reactions such as allergies, with symptoms occurring either directly after injection or delayed by several months or years [2–8]. To date, the triggers of allergic reactions remain largely unknown [4, 7, 9, 10]. As soluble parts of the inks are excreted from the tattooed site within short time, additives in inks such as preservatives are not considered to be involved in delayed-type allergic reactions [11]. However, the persistent colorants remain in the skin, thus presenting a possible source for allergens. Recent investigations have shown high prevalence of so-called naphthol AS (NAS) pigments in skin biopsies of allergic tattoo reactions [11, 12]. These azo pigments are synthesized by azo coupling with derivatives of 2-hydroxy-3-naphthanilide (naphthol AS) as coupling component [13]. In a study with 104 allergic reactions to red tattoo color, NAS pigments were detected in 55% of the cases, suggesting that they might be involved in allergy triggering mechanisms [11]. Due to the delay in symptoms, it is proposed that the pigments undergo a (slow) haptenization process [7, 10]. This requires an initial enzyme-mediated or abiotic activation step, potentially transforming the structure to a reactive electrophile. Afterwards, specific binding to protein structures in the skin may lead to the formation of hapten-protein conjugates that are able to initiate sensitization [11, 14–16].

However, the enzymatic metabolism of pigments at the molecular level has hardly been studied and the role of enzymes in tattooed skin remains largely unknown [17]. The largest group of metabolizing enzymes responsible for oxidative processes in the skin are cytochrome P450 monooxygenases (CYP450). Aldehyde oxidases or dehydrogenases may also play a role [16, 18, 19]. The main concentration of CYP450 is localized in keratinocytes of the epidermis, which should not be relevant in tattoo pigment metabolism, as pigments are deposited in the upper dermis. Cells of the dermis also show enzymatic activity, although weaker than in the epidermal layer [18–20]. Despite their low solubility, the extraordinarily long exposure time might make pigments susceptible to slow biotransformation processes in the dermis. Furthermore, active and passive transport of pigments leads to the deposition of pigments in local lymph nodes [21–23] and it is assumed that pigments partly become systemically available and are transported to highly metabolizing organs such as the liver [2, 17, 22]. A sole approach to modelling the oxidation of pigments has been described by Cui *et al*. for the azo pigment Pigment Yellow 74 (PY 74) by human and rat CYP450. In this study, the azo pigment underwent single hydroxylation and *O*-demethylation [20]. Despite that, models for the investigation of biotransformation of pigments are scarce and complicated by the low solubility and thus availability for the model systems. Furthermore, a major drawback in pigment research is the lack of clean standards. This is also reflected in the used pigments in inks, because most often the pigments applied for tattooing are not intended for intradermal application but originate from pigment manufacturers producing for industrial purposes, such as the paint or printing ink industries [1, 6, 24]. During synthesis, precipitation and little need for cleanup result in a purity of less than 90% and impurities such as synthesis by-products and precursors are often present in finished formulations. The particles are furthermore coated with various chemicals to achieve the desired technical properties of pigments in their intended application [1].

Electrochemistry (EC) has been proven to be a fully instrumental, fast, and easy-to-use approach to simulating the metabolism of xenobiotics, thus complementing *in vitro* and *in vivo* assays. The basic principle is using electrochemically induced redox reactions to mimic the behavior of oxidizing and reducing enzymes in the human body. Clean standards are injected into an electrochemical cell, oxidized or reduced and the electrochemically formed transformation products (TPs) are often investigated by direct hyphenation to mass spectrometry (MS). The instant detection approach allows to monitor even the formation of highly reactive substances. A further advantage of EC-MS is the compatibility with organic solvents. This especially benefits the accessibility of pigments with low aqueous solubility for metabolism simulation via EC-MS, compared to conventional *in vitro* experiments often conducted in aqueous media. For various drugs, TPs similar to those found by CYP450 family-based conversions have been identified using this approach [25–29]. Accordingly, EC-MS has already been applied for mimicking the metabolism of skin sensitizing agents such as *para*-phenylenediamine (PPD) and eugenol [30–32]. After EC transformation, on- or offline-coupling of liquid chromatography (LC) allows for the separation of TPs and potential isomers formed in the electrochemical cell [33–38]. Changing positions, by coupling the EC-cell to the column effluent, EC can be used as a tool for ionization enhancement of naturally non- or poorly ionizable substances [39, 40]. Similarly, this LC-EC-MS setup allows for the investigation of complex mixtures, as for example shown for protein digests [41].

**Fig. 1:**
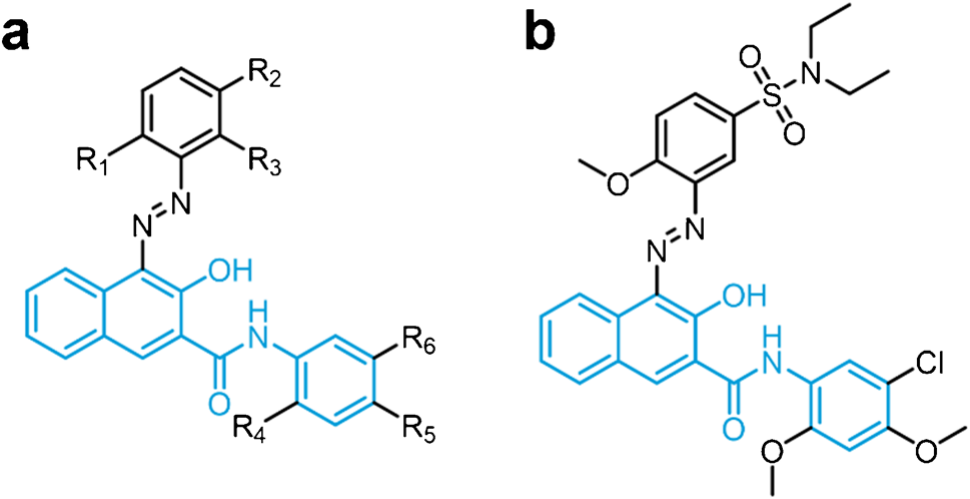
General structure of naphthol AS pigments (**a**) and Pigment Red 5 (PR 5) (**b**). The core structure naphthol AS (2-hydroxy-3-naphthanilide) is marked in blue

Considering that commercially available pigments are not available as clean standards but as complex mixtures with various impurities, this work focuses on the development of an online-LC-EC-MS approach for simulation of the metabolism of a red (tattoo) pigment. For this purpose, Pigment Red 5 (PR 5) from the group of NAS pigments, which have frequently been identified in allergic skin reactions, is investigated [11, 12]. Substituents such as three methoxy groups, one chlorine, and a sulfonamide (-SO_2_N(C_2_H_5_)_2_) group activate the pigment towards electrochemical conversion. The general structure of NAS pigments and of PR 5 is depicted in Fig. 1 [13]. To demonstrate the variety of impurities in commercially available pigments, two different PR 5 samples were investigated. The impurity profiles were characterized by online-LC-high-resolution-MS as well as high-resolution-tandem-MS (MS/MS). The developed separation was then used for online-LC-EC-MS to electrochemically oxidize and reduce the isolated pigment. Characterization of transformation products was carried out by high-resolution-MS and -MS/MS.

## Experimental

### Pigment preparation

Two different PR 5 samples were investigated. PR 5a was provided within a collaboration with the Cantonal Laboratory Basel-City (Basel, Switzerland), while PR 5b was obtained directly from a pigment manufacturer in Germany. For screening for impurities via LC-MS, 6.3 mg of each pigment was suspended in 10 mL acetonitrile (ACN, VWR International, Radnor, PA, USA) to obtain a saturated solution. The supernatant solution was filtered through polytetrafluoroethylene (PTFE) syringe filters (pore size 0.2 μm, VWR International) and diluted by mixing 100 µL of the filtrate with 900 µL of 5 mM ammonium formate/ACN (50/50, *v*/*v*) prior to injection. Ammonium formate was obtained from Sigma-Aldrich (Steinheim, Germany). For LC-EC-MS analysis, a washing step was implemented to reduce the intensity of highly soluble and easily ionizable by-products. This allowed higher concentrations of the solution to be injected, while avoiding the overload of the detector with by-products. The pigments were again prepared as a saturated solution in ACN, centrifuged at 3000 rpm for 10 min, and the washing liquid was discarded. The precipitate was redissolved, filtered through PTFE syringe filters, and diluted by mixing 150 µL of the filtrate with 850 µL of 5 mM ammonium formate/ACN (50/50, *v*/*v*).

### Liquid chromatography

Chromatography was performed using an Ultimate 3000 UHPLC (Thermo Fisher Scientific, Germering, Germany) equipped with a SRD-3400 degasser, HPG-3200RS pump, WPS-3000RS autosampler, and TCC-2000RS column oven. As stationary phase, a C18 reversed-phase (RP) column was used (Xbridge, 2.1 × 100 mm, 3.5 µm particles, Waters, Eschborn, Germany). Solvent A was 5 mM ammonium formate (pH 7), solvent B was ACN, the flow rate was set to 300 µL min^−1^, and a volume of 5 µL was injected. Gradient elution was performed with a linear gradient from 50 to 95% B in 12 min, keeping 100% B for 3 min, followed by a re-equilibration step of 50% B for another 3 min. The column oven temperature was 40 °C. Blank runs in between every pigment sample were implemented to clean the column from remaining pigment.

### Electrochemistry

For the LC-EC-MS experiments, the effluent of the column was directly coupled to an electrochemical thin-layer cell (µ-PrepCell 2.0, Antec Scientific, Alphen aan den Rijn, The Netherlands). Boron-doped diamond (BDD) was used as a working electrode while the counter electrode was made of titanium. The potential was controlled using a pseudo-reference Pd/H_2_ electrode and an in-house developed potentiostat operated by in-house written software. The potential was set to a constant value over the whole LC-EC-MS run, depending on desired oxidative or reductive conditions. The used potentials were 0.0 V, 0.5 V, 1.0 V, 1.5 V, 2.0 V, 2.5 V, and 3.0 V vs Pd/H_2_ for oxidation and −0.0 V, −0.5 V, −1.0 V, −1.5 V, −2.0 V, and −2.5 V vs Pd/H_2_ for reduction.

### Mass spectrometry

All experiments were performed on a 6530 Q-TOF LC-MS mass spectrometer (Agilent Technologies, Santa Clara, CA, USA) equipped with a Jet Stream Technology ion source (AJS, Agilent Technologies), and controlled by MassHunter Workstation software (V.10.1, Agilent Technologies). Detailed source and MS parameters can be found in the supporting information (SI). The analyses were run in data-dependent acquisition mode (DDA) with collision energies of 10, 20, 30, 40, and 50 eV, respectively, and a quadrupole isolation window of 1.3 mass-to-charge ratio (*m*/*z*). Spectra were acquired in both polarities in the mass range of 100–1500 *m*/*z* in MS and 20–700 *m*/*z* in MS/MS mode, with a spectra rate of 4 Hz for MS and 5 Hz for MS/MS. Data was analyzed using Qualitative Analysis (V.10.0, Agilent Technologies) and the open-source software tools MZmine 3 [42] and SIRIUS [43].

## Results and discussion

### Impurities in two different PR 5 samples

**Fig. 2:**
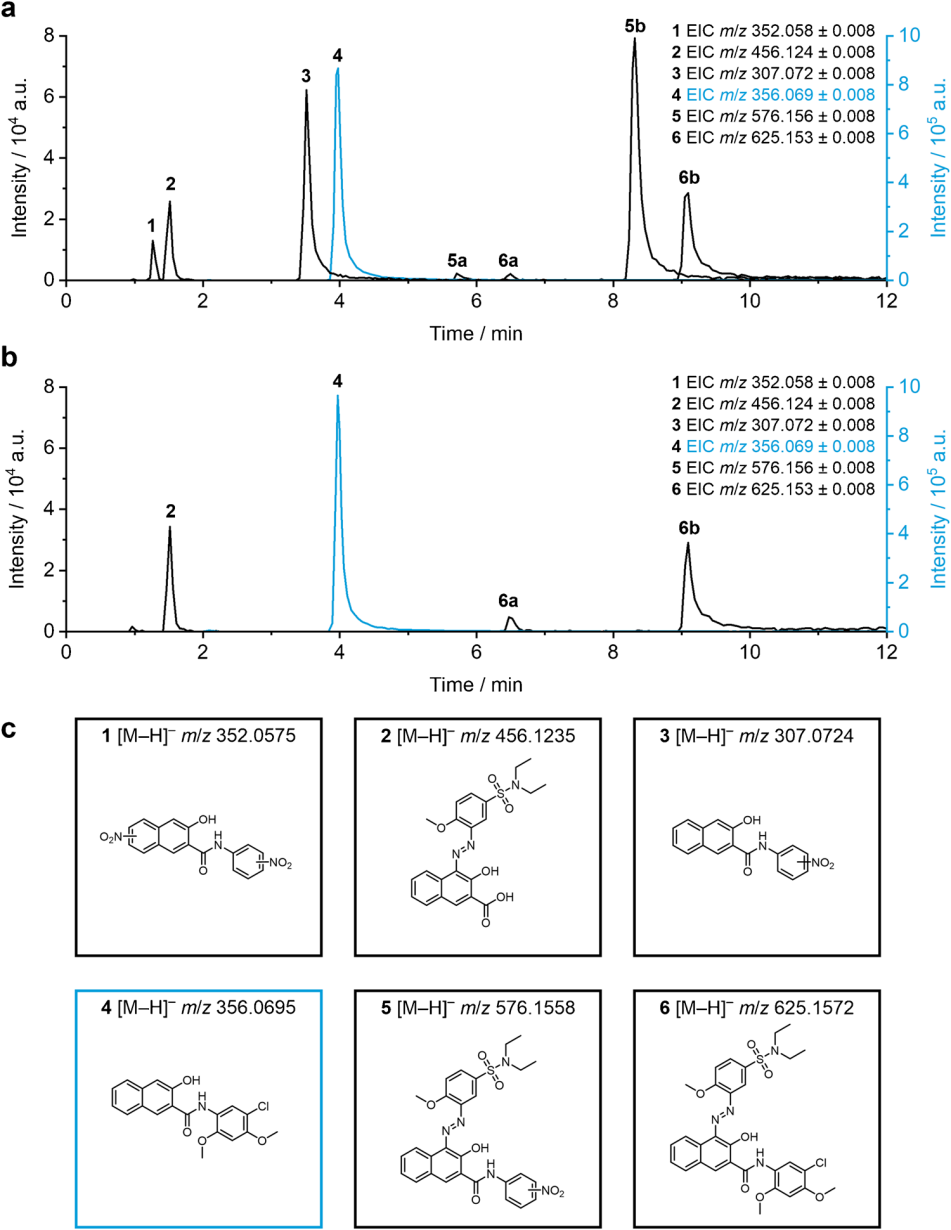
Extracted ion chromatograms (EICs) of PR 5 (*m*/*z* 625.153, **6**) and exemplarily observed impurities in PR 5a (**a**) and PR 5b (**b**) obtained by LC-MS in negative ion mode. Due to high intensity, *m*/*z* 356.069 (**4**) uses the blue *y*-axis on the right for intensity. Displayed structural proposals (**c**) are based on MS/MS experiments (see Table S2-1 in SI). Substitution patterns for PR 5 and its synthesis precursor are depicted according to Hunger and Schmidt [13]. The position of the NO_2_ substitutions cannot be determined unequivocally based on the acquired data

One major drawback in research targeting pigment metabolism is the lack of availability of clean standards. The potential presence of synthesis precursors and synthesis by-products hampers classical approaches and metabolism simulation by direct EC-MS, as the TPs cannot unambiguously be linked to the pigment but might be generated from a variety of impurities. For this reason, an impurity profiling was conducted to characterize the two obtained pigments. The LC-MS setup revealed the presence of multiple by-products and a synthesis precursor in both samples. Figure 2 displays exemplary extracted ion chromatograms (EICs) of *m*/*z* detected in sample PR 5a (Fig. 2a) and PR 5b (Fig. 2b) in the negative ion mode. For the *m*/*z* of PR 5 (*m*/*z *625.153), two separate peaks can be observed (*t*_R_ = 6.5 and 9.1 min) which most likely result from *cis* and *trans* isomeric azo forms. Both samples furthermore contain *m*/*z* 356.069, presenting a characteristic chlorine isotope pattern. This *m*/*z* can be assigned to a NAS derivative, which is needed as a coupling component for azo coupling during synthesis of PR 5. The modified NAS can be detected with very high intensity, due to improved solubility and potentially higher ionization efficiency. Another shared impurity in both samples is *m*/*z* 456.124, which likely contains a carboxylic acid group instead of the anilide. This could either be formed by hydrolysis of the amide or be present as a remnant from the coupling component synthesis, followed by azo coupling. Noteworthy, PR 5a also contains another NAS pigment (*m*/*z* 576.156), substituted with a nitro group at the anilide, instead of two methoxy groups and one chlorine as in PR 5. Comparable to the *m*/*z* of PR 5, the second NAS pigment shows two peaks, potentially resulting from *cis*/*trans* azo isomers. Likewise, the corresponding azo coupling component, NAS substituted with one nitro group (*m*/*z* 307.072), is present in PR 5a. Both NAS pigments (*m*/*z *625.153 and *m*/*z* 576.156) show tailing effects on the RP column, which could be a result of their poor solubility, leading to precipitation on the column and slow redissolution in the mobile phase.

Tentative structures for the discussed *m*/*z* values are proposed in Fig. 2c based on MS/MS data. The corresponding fragments, including proposed ion formulae and mass deviations, are listed in the SI. Structures related to PR 5 (*m/z* 625.153) displayed in Fig. 2c are depicted with the substitution pattern described in Hunger and Schmidt [13]. The position of NO_2_ substitutions cannot be determined unequivocally by MS/MS analysis. The SI encloses a list of further detected features in both polarities. This includes proposed ion formulae, calculated *m*/*z*, and mass deviations for precursor and fragments in both pigment samples. While some features are detected in both samples, others are only present in one. This showcases the distinct impurity profiles present in different samples of PR 5. Overall, the pigment was successfully isolated from occurring by-products so that the obtained separation was used for all further LC-EC-MS experiments.

### Online-LC-EC-MS

**Fig. 3:**
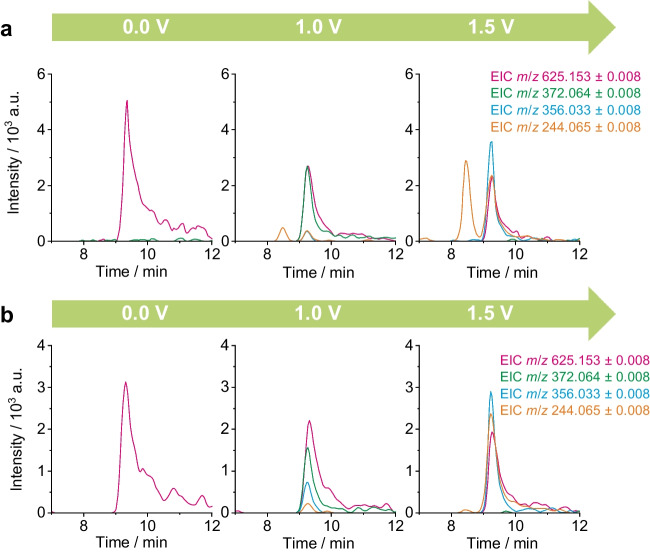
Extracted ion chromatograms (EICs) of PR 5 (*m*/*z* 625.153) and its oxidative transformation products observed at the same retention time, obtained by online-LC-EC-MS in the negative ion mode. Oxidative runs at 0.0 V, 1.0 V, and 1.5 V are shown for the samples PR 5a (**a**) and PR 5b (**b**), respectively

After the separation of the pigment from its impurities was achieved, the electrochemical cell was inserted between LC and MS and consecutive runs with different potentials were carried out. The experiments were conducted for negative and positive ion mode, respectively. Figure 3 shows the EICs of PR 5 (*m*/*z* 625.153) in the LC-EC-MS runs in negative ion mode for both PR 5 samples at increasing oxidative potentials. Compared to the LC-MS experiments, peak broadening and tailing of *m*/*z* 625.153 are observed in LC-EC-MS analyses. This can partly be explained by the introduced dead volume post-column. Furthermore, pigments present large, conjugated π-electron systems, so that surface adsorption to the electrodes material can also contribute to peak broadening. Figure 3 shows a decrease in intensity of the pigment signal upon increasing the applied potential, which indicates an oxidative electrochemical transformation. By filtering for newly occurring features in the retention time window of the pigment in MZmine, three main oxidative transformation products (OTPs) of PR 5 are observed at the selected potentials. Figure 3 depicts the runs with 0.0 V, 1.0 V, and 1.5 V, as they exhibit the highest intensity for the main OTPs. By applying 1.0 V, *m*/*z* 372.064 is formed at the pigment’s retention time, but disappears at the higher potential run of 1.5 V. At this voltage, the other two OTPs *m*/*z* 356.033 and *m*/*z* 244.065 become more intense. At 0.5 V, no products were yet detected. After applying potentials ≥2.0 V, the intensity of all TPs decreases until they are no longer detectable at 3.0 V. As visible in Fig. 3, both PR 5 samples lead to the formation of the same OTPs, although they showed different impurity profiles in the previous experiments. For *m*/*z *244.065, it can be observed that it is not only formed by PR 5, but also by the second NAS pigment detected in PR 5a at *t*_R_ = 8.5 min. This highlights the importance of the online separation approach, because classical EC-MS setups without previous chromatographic separation are not able to distinguish the corresponding origin of the TP. The same OTP with *m*/*z* 244.065 gives further hints for a structural resemblance of both precursors. In addition to the three OTPs in negative ion mode, two OTPs were detected in positive ion mode. All observed OTPs are summarized in Table 1 and ion formulae are proposed.

**Table 1:** List of transformation products (TPs) of Pigment Red 5 (PR 5) detected in negative and positive ion mode and assigned to their corresponding parent compound by retention time filtering. TPs were detected in both PR 5 samples. The potential with highest intensity of the TP is listed and ion formulae, including calculated *m*/*z* and mass deviation, are proposed. TPs are named by the electrochemical conditions (oxidative = OTP, reductive = RTP), the polarity (+/−) in which they were observed, and their respective nominal *m*/*z*

Potential / V	Recorded *m*/*z*	Ion formula	Calculated *m*/*z*	Δ* m*/*z* / ppm	Name
Negative ion mode					
+1.0 (Ox)	372.0647	C_19_H_15_ClNO_5_^−^	372.0644	0.7	OTP(−)372
+1.5 (Ox)	356.0334	C_18_H_11_ClNO_5_^−^	356.0331	0.8	OTP(−)356
+1.5 (Ox)	244.0651	C_10_H_14_NO_4_S^−^	244.0649	0.8	OTP(−)244
−2.0 (Red)	371.0807	C_19_H_16_ClN_2_O_4_^−^	371.0804	0.8	RTP(−)371
Positive ion mode					
+2.0 (Ox)	358.0463	C_18_H_13_ClNO_5_^+^	358.0477	−3.9	OTP(+)358
+2.0 (Ox)	244.1008	C_11_H_18_NO_3_S^+^	244.1002	2.5	OTP(+)244
−2.0 (Red)	259.1118	C_11_H_19_N_2_O_3_S^+^	259.1110	3.1	RTP(+)259

**Fig. 4:**
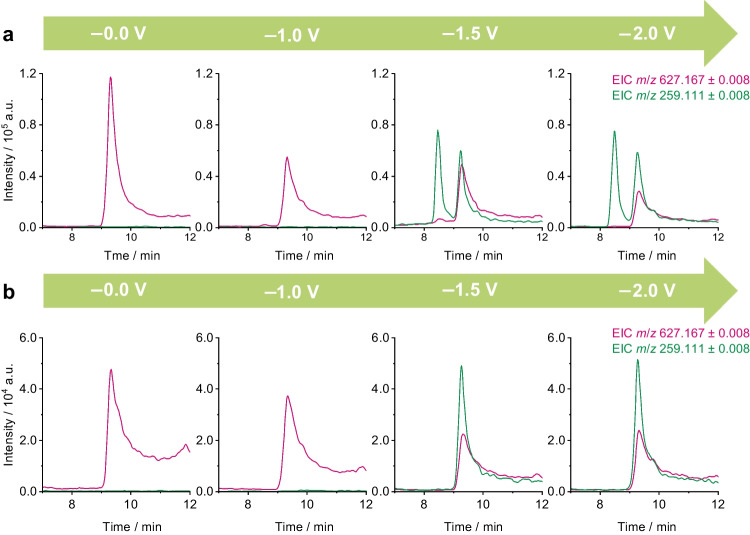
Extracted ion chromatograms (EICs) of PR 5 (*m*/*z* 627.167) and its reductive transformation product observed at the same retention time, obtained by online-LC-EC-MS in the positive ion mode. Reductive runs at −0.0 V, −1.0 V, −1.5 V, and −2.0 V are shown for the samples PR 5a (**a**) and PR 5b (**b**), respectively

Similar to the oxidative investigation, Fig. 4 shows the electrochemical reduction in positive ion mode for PR 5a and PR 5b from 0.0 to −2.0 V. In this polarity and electrochemical condition, one main reductive TP (RTP) can be observed at *m*/*z* 259.111. The formation starts in the run with −1.5 V applied to the cell and remains with similar intensities at −2.0 V and −2.5 V. Again, the RTP forms in both PR 5 samples and can be linked to the pigment by the retention time. Moreover, in PR 5a, it also appears at the retention time of the second NAS pigment at *t*_R_ = 8.5 min, once more highlighting a structural resemblance of the precursors. In both samples, a second RTP of PR 5 was observed in the negative ion mode.

The observed OTPs and RTPs in both polarities are summarized in Table 1. The TPs are named by the electrochemical conditions (oxidative = OTP, reductive = RTP), the polarity (+/−) in which they were observed, and their respective nominal *m*/*z*. Table 1 includes the recorded *m*/*z*, proposed ion formulae, calculated *m*/*z*, and mass deviations.

**Fig. 5:**
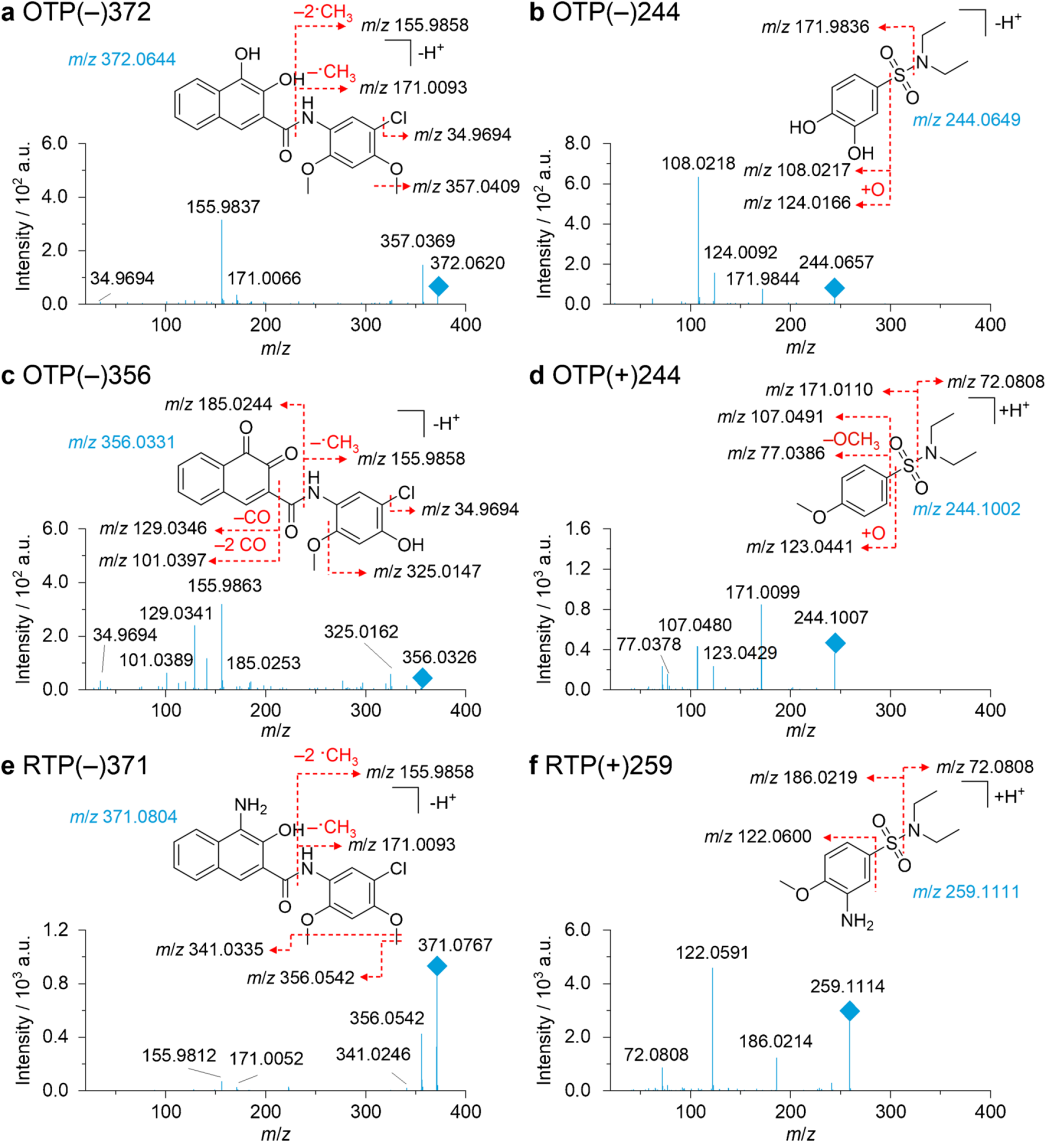
MS/MS spectra of oxidative (OTP) and reductive (RTP) transformation products of PR 5 in negative and positive ion mode obtained by online-LC-EC-MS/MS analysis. TPs are named by the electrochemical conditions (oxidative = OTP, reductive = RTP), the polarity (+/−) in which they were observed, and their respective nominal *m*/*z*

To further characterize the TPs, MS/MS spectra were recorded and structural annotations were proposed to understand the oxidative and reductive transformation pathways taking place in the electrochemical cell. Figure 5 shows the MS/MS of all listed TPs in Table 1, except for OTP(+)358, which corresponds to OTP(−)356 in positive polarity and can be found in the SI.

### Electrochemical transformation pathways

**Fig. 6:**
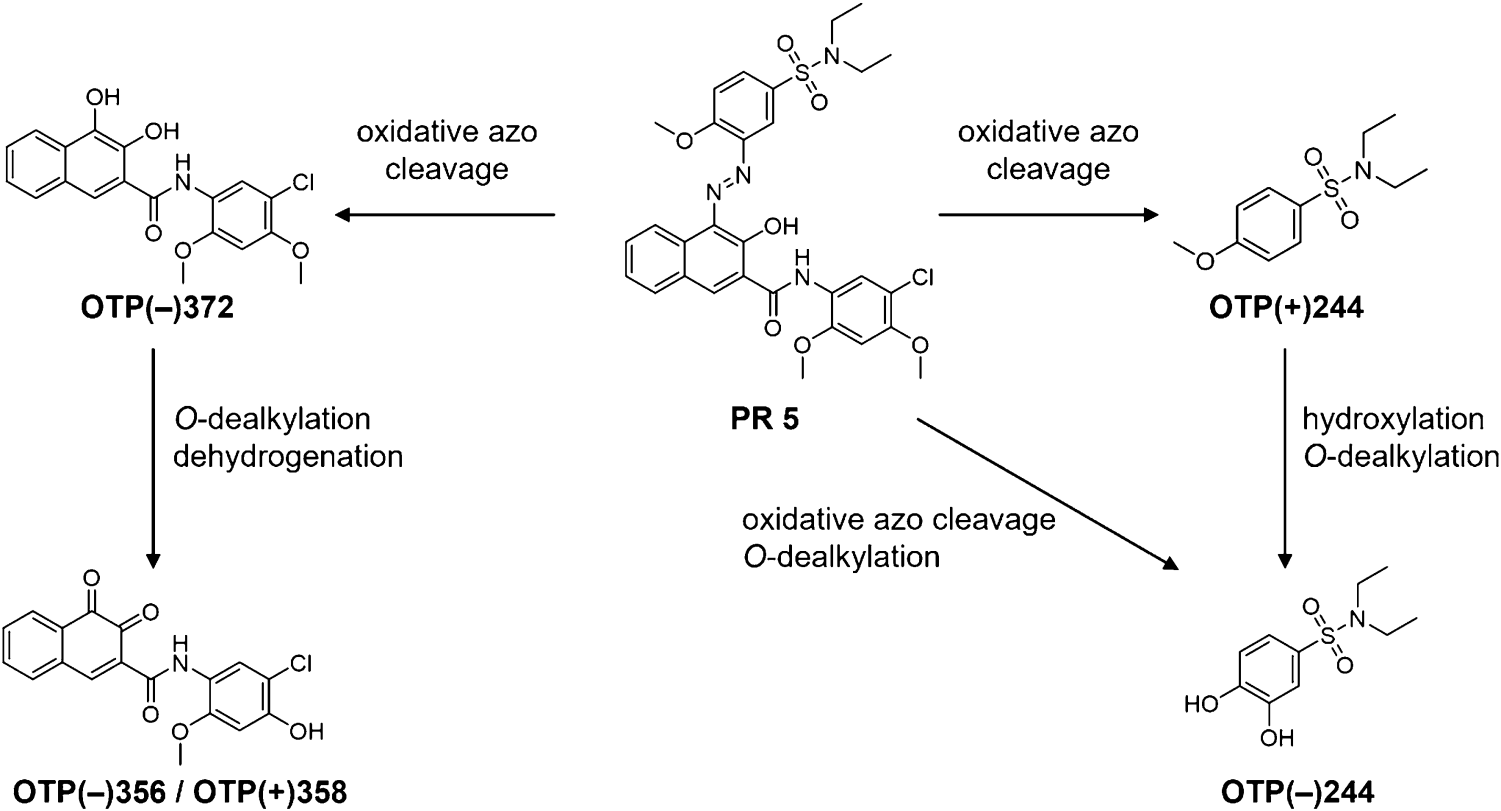
Oxidative electrochemical transformation routes of Pigment Red 5 (PR 5) observed by online-LC-EC-MS. Oxidative transformation products (OTPs) are named by their observed polarity (+/−) and their respective nominal *m*/*z*. Substitution patterns are displayed according to the initial substitution in PR 5, according to Hunger and Schmidt [13] and the most likely position for hydroxylation. The *O*-demethylation position in OTP(−)356 / OTP(+)358 cannot be determined unequivocally based on the acquired data

**Fig. 7:**
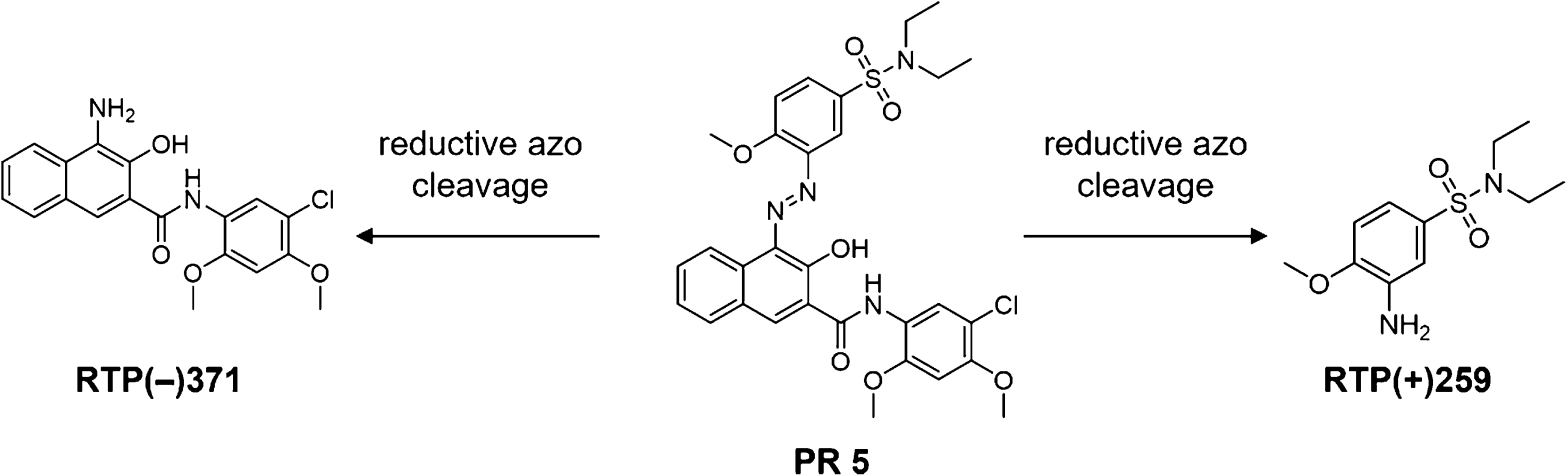
Reductive electrochemical transformation routes of Pigment Red 5 (PR 5) observed by online-LC-EC-MS. Reductive transformation products (RTPs) are named by their observed polarity (+/−) and their respective nominal *m*/*z*. Substitution patterns are displayed according to the initial substitution in PR 5, according to Hunger and Schmidt [13]

The proposed structures and derived electrochemical transformation pathways are depicted in Fig. 6 for oxidation and Fig. 7 for reduction. In oxidative electrochemical conditions, the azo linkage of the pigment is oxidatively cleaved. Enzymatic cleavage by CYP450 has already been described for soluble azo dyes, such as Sudan 1, yielding ring-hydroxylated metabolites [44]. Due to the low observed potential for the formation of hydroxylated products in this study (1.0 V), it is likely that the electrochemical oxidation is initiated by single-electron-transfer, although the formation of hydroxy radicals is possible on BDD at higher potentials. The site of the initial single-electron-oxidation is dependent on the substitution pattern and thus the activation of the respective rings. For PR 5, OTP(+)244 and OTP(−)372 are observed, indicating an initial single-electron-oxidation at the naphthol ring followed by addition of water and cleavage of the C-N bond. OTP(−)244 may indicate a competing activation of the oxidative azo-cleavage by single-electron-oxidation at the sulfonamide substituted ring. It can, however, also be derived from OTP(+)244 by hydroxylation and *O*-dealkylation. A further oxidation of OTP(−)372 through *O*-dealkylation and dehydrogenation leads to the formation of an *ortho*-quinone (OTP(−)356). The *O*-dealkylation of methoxy groups in pigments has also been observed by CYP450 enzymes for PY 74 [20]. For OTP(−)356, it has to be noted, that the dealkylation position cannot be determined unequivocally, as the precursor contains two methoxy groups for possible dealkylation that cannot be differentiated in the MS/MS data.

Under reductive electrochemical conditions, primary aromatic amines (PAAs) are formed from PR 5 by reductive azo cleavage (RTP(−)371 and RTP(+)259). This observation is comparable to electrochemical reduction targeting soluble azo dyes and enzymatic cleavage of azo dyes by azoreductases [45–47]. Several PAAs have also been identified in photochemical cleavage studies of pigments [17, 48]. A subsequent oxidative dehydrogenation of the amino and the adjacent hydroxy group could further lead to the formation of an *ortho*-quinone imine.

Future investigations need to show if similar (enzymatic) reduction and oxidation pathways of PR 5 can take place in the skin. Further biotransformation steps, such as amide hydrolysis in the anilide moiety, not mimicable by EC, cannot be excluded to be happening *in vivo*. With regard to the elicitation of allergies, quinones and quinone imines have been shown to exhibit sensitization potential in previous studies. Depending on their structure, their electrophilic properties can lead to the formation of protein conjugates [49, 50]. To explore the individual reactivity of the here presented TPs, targeted synthesis followed by conjugation experiments to skin proteins could be promising. This could be a first step towards a better understanding of tattoo related allergies.

The previously described retention time filtering for TPs and subsequent structural annotation was applied to selected impurities (see SI-3). A special focus was directed to the second NAS pigment observed in PR 5a. The observed electrochemical transformation routes show strong resemblance to the ones observed for PR 5. Table S3-1 holds all observed TPs including proposed ion formulae, calculated *m*/*z*, and mass deviation in both polarities for the second NAS pigment. MS/MS spectra and proposed transformation pathways are displayed in Fig. S3-3, Fig. S3-5, and Fig. S3-6. In oxidative conditions, oxidative azo cleavage is followed by quinone formation and further hydroxylation. The application of reductive potentials yields corresponding PAAs that are generated by reductive azo cleavage. The results indicate that the presented electrochemical transformation routes are applicable to other NAS pigments.

## Conclusions

Little is known about the metabolism of tattoo pigments in human skin and investigations of biotransformation of hardly soluble and impure substances are difficult to perform. Thus, an online-LC-EC-MS approach was developed to simulate the metabolism of the red organic azo pigment PR 5. The concept of online chromatographic separation prior to electrochemical transformation was demonstrated by studying two PR 5 samples with individual impurity profiles. Although different impurities were identified for each sample, the same TPs for PR 5 were observed for both pigment formulations using the online-LC-EC-MS approach. Distinct retention times enabled the assignment of the TPs to their corresponding parent compound and allow to work with standards of limited purity such as commercially available pigments for electrochemical metabolism simulation. For the tattoo pigment PR 5, oxidative azo cleavage was observed, followed by quinone formation due to the hydroxy substitution on the naphthol ring. In reductive mode, reductive azo cleavage resulting in primary aromatic amine formation was simulated. The presented method addresses the data gaps on pigment stability towards oxidative or reductive conditions and potential metabolism in human skin and the following excretory routes after tattooing. The purely instrumental approach grants mechanistic insight and transformation pathways are proposed, paving the way for a more targeted approach in future investigations, for example, using enzymatic cleavage. In this way, the formed electrochemical TPs can facilitate the search for potential allergens formed from naphthol AS pigments.

## Supplementary Information

Below is the link to the electronic supplementary material.

Supplementary file1 (DOCX 1.08 MB)
