# Supplementary material for: Working with technical purity: simulation of red tattoo pigment metabolism by online-liquid chromatography-electrochemistry-mass spectrometry
Source: Anal Bioanal Chem. 2025 Jan 4;418(2):655–65. doi: 10.1007/s00216-024-05709-8 (PMC12783158; doi:10.1007/s00216-024-05709-8)
Supplement: Supplementary file 1 — Supplementary file1 (DOCX 1.08 MB) [file 216_2024_5709_MOESM1_ESM.docx]

Supporting Information

**Working with technical purity: Simulation of red tattoo pigment metabolism by online-liquid chromatography-electrochemistry-mass spectrometry**

Carina Wolf^a^, Franziska Krall^a^, Valentin Göldner^a,b^, Uwe Karst^a^

^a^Institute of Inorganic and Analytical Chemistry, University of Münster, Münster, Germany
^b^Department of Environmental Geosciences, Centre for Microbiology and Environmental Systems Science, University of Vienna, Vienna, Austria

Corresponding author: Carina Wolf: carina.wolf@uni-muenster.de

Journal: Analytical and Bioanalytical Chemistry

Contents

[1 Additional experimental parameters 2](#_Toc185837649)

[1.1 MS parameters 2](#_Toc185837650)

[2 Impurity profiling of two different PR 5 samples 3](#_Toc185837651)

[3 LC-EC-MS 7](#_Toc185837652)

[3.1 Electrochemical transformation of PR 5 7](#_Toc185837653)

[3.2 Electrochemical transformation of selected byproducts 7](#_Toc185837654)

[3.2.1 Impurity C_28_H_27_N_5_O_7_S, PR 5a 7](#_Toc185837655)

[3.2.2 Impurity C_19_H_16_ClNO_4_, PR 5a and PR 5b 13](#_Toc185837656)

[3.2.3 Impurity C_17_H_12_N_2_O_4_, PR 5a 15](#_Toc185837657)

# Additional experimental parameters

## MS parameters

Table S1‑1 Source and MS TOF parameters of 6530 Q-TOF mass spectrometer

| **Source** | |
| --- | --- |
| Gas Temperature / °C | 300 |
| Dry gas / L min^−1^ | 8 |
| Nebulizer / psi | 35 |
| Sheath gas temperature / °C | 350 |
| Sheath gas flow / L min^−1^ | 11 |
| VCap / V | 3500 |
| Nozzle voltage / V | 1000 |
| **MS TOF parameters** | |
| Fragmentor / V | 175 |
| Skimmer / V | 65 |
| Oct1 RF / Vpp | 750 |

# Impurity profiling of two different PR 5 samples

For evaluation of the LC-MS analysis, the runs of the pigment samples were blank subtracted and the remaining features were investigated. Both PR 5 samples contained multiple features additionally to the pigment mass. Features that could be assigned to a plausible ion formula are listed in Table S2-1 for negative ion mode and Table S2-2 for positive ion mode. The tables hold recorded *m*/*z* and retention time of the pigment and exemplary impurities, and their occurrences in sample a and b. Ion formulae and calculated *m*/*z,* including mass deviation of precursor and corresponding fragments are listed. Structural proposals for six features in the negative ion mode were derived from the MS/MS data and are displayed in the main document.

Table S2‑1 Detected features in LC-MS analysis of both Pigment Red 5 samples in the negative ion mode, which could be assigned to a plausible ion formula. The occurrence in the respective sample (a,b), their retention time (*t_R_*), and recorded *m*/*z* of the precursor are listed. Ion formulae are proposed, including calculated *m*/*z* and mass deviation. Furthermore, recorded fragments including proposed ion formulae, calculated *m*/*z*, and mass deviation are contained

| **Sample** | ***t_R_*** | **Precursor** | | | | **Fragments** | | | |
| --- | --- | --- | --- | --- | --- | --- | --- | --- | --- |
|  |  | **Recorded *m*/*z*** | **Ion formula** | **Calculated *m*/*z*** | **Δ *m*/*z* / ppm** | **Recorded *m*/*z*** | **Ion formula** | **Calculated *m*/*z*** | **Δ *m*/*z* / ppm** |
| a | 1.22 | 401.0555 | C_19_H_14_ClN_2_O_6_^−^ | 401.0546 | 2.3 | 386.0309 | C_18_H_11_ClN_2_O_6_^−^ | 386.0311 | −0.6 |
|  |  |  |  |  |  | 369.0284 | C_18_H_10_ClN_2_O_5_^−^ | 369.0284 | 0.1 |
|  |  |  |  |  |  | 187.0271 | C_10_H_5_NO_3_^−^ | 187.0275 | −2.1 |
|  |  |  |  |  |  | 155.9852 | C_6_H_3_ClNO_2_^−^ | 155.9858 | −3.7 |
| a | 1.27 | 352.0583 | C_17_H_10_N_3_O_6_^−^ | 352.0575 | 2.2 | 322.0595 | C_17_H_10_N_2_O_5_^−^ | 322.0595 | −0.1 |
|  |  |  |  |  |  | 306.0644 | C_17_H_10_N_2_O_4_^−^ | 306.0646 | −0.7 |
|  |  |  |  |  |  | 188.0346 | C_10_H_6_NO_3_^−^ | 188.0353 | −3.8 |
|  |  |  |  |  |  | 158.0388 | C_10_H_6_O_2_^−^ | 158.0373 | 9.3 |
|  |  |  |  |  |  | 45.9941 | NO_2_^−^ | 45.9935 | −14.1 |
| a,b | 1.52 | 456.1230 | C_22_H_22_N_3_O_6_S^−^ | 456.1235 | −1.1 | 412.1312 | C_21_H_22_N_3_O_4_S^−^ | 412.1337 | −5.9 |
|  |  |  |  |  |  | 212.0735 | C_10_H_14_NO_2_S^−^ | 212.0751 | −7.4 |
|  |  |  |  |  |  | 139.9929 | C_6_H_4_O_2_S^−^ | 139.9937 | −6.1 |
|  |  |  |  |  |  | 136.044 | C_4_H_10_NO_2_S^−^ | 136.0438 | 1.7 |
| a | 2.30 | 301.0581 | C_13_H_9_N_4_O_5_^−^ | 301.0578 | 0.9 | 137.0352 | C_6_H_5_N_2_O_2_^−^ | 137.0357 | −3.3 |
|  |  |  |  |  |  | 107.0361 | C_6_H_5_NO^−^ | 107.0377 | −14.6 |
|  |  |  |  |  |  | 45.9927 | NO_2_^−^ | 45.9935 | −16.3 |
| a | 3.51 | 307.0723 | C_17_H_11_N_2_O_4_^−^ | 307.0724 | −0.4 | 261.0782 | C_17_H_11_NO_2_^−^ | 261.0795 | −5.1 |
|  |  |  |  |  |  | 143.0497 | C_10_H_7_O^−^ | 143.0502 | −3.8 |
|  |  |  |  |  |  | 137.0348 | C_6_H_5_N_2_O_2_^−^ | 137.0357 | −6.2 |
|  |  |  |  |  |  | 115.0548 | C_9_H_7_^−^ | 115.0553 | −4.6 |
|  |  |  |  |  |  | 45.9932 | NO_2_^−^ | 45.9935 | −5.5 |
| a,b | 3.97 | 356.0696 | C_19_H_15_ClNO_4_^−^ | 356.0695 | 0.3 | 341.0463 | C_18_H_12_ClNO_4_^−^ | 341.0460 | 0.8 |
|  |  |  |  |  |  | 326.0223 | C_17_H_9_ClNO_4_^−^ | 326.0226 | −0.8 |
|  |  |  |  |  |  | 171.0093 | C_7_H_6_ClNO_2_^−^ | 171.0093 | 0.3 |
|  |  |  |  |  |  | 155.9860 | C_6_H_3_ClNO_2_^−^ | 155.9858 | 1.4 |
|  |  |  |  |  |  | 34.9685 | Cl^−^ | 34.9694 | −25.8 |
| Continued on next page | | | | | | | | | |
| Table S2-1 - continued from previous page | | | | | | | | | |
| **Sample** | ***t_R_*** | **Precursor** | | | | **Fragments** | | | |
|  |  | **Recorded *m*/*z*** | **Ion formula** | **Calculated *m*/*z*** | **Δ *m*/*z* / ppm** | **Recorded *m*/*z*** | **Ion formula** | **Calculated *m*/*z*** | **Δ *m*/*z* / ppm** |
| a,b | 6.29 | 597.1462 | C_30_H_30_ClN_2_O_7_S^−^ | 597.1468 | −1.0 | 582.1173 | C_29_H_27_ClN_2_O_7_S^−^ | 582.1233 | −10.3 |
|  |  |  |  |  |  | 565.1174 | not identified | / | / |
|  |  |  |  |  |  | 550.0933 | not identified | / | / |
|  |  |  |  |  |  | 535.0699 | not identified | / | / |
|  |  |  |  |  |  | 352.0995 | C_20_H_18_NO_3_S^−^ | 352.1013 | −5.1 |
|  |  |  |  |  |  | 212.0147 | not identified | / | / |
|  |  |  |  |  |  | 155.9852 | C_6_H_3_ClNO_2_^−^ | 155.9858 | −3.7 |
| a,b | 6.65 | 725.1456 | C_39_H_31_Cl_2_N_2_O_8_^−^ | 725.1463 | −1.0 | 356.0690 | C_19_H_15_ClNO_4_^−^ | 356.0695 | −1.4 |
|  |  |  |  |  |  | 341.0460 | C_18_H_12_ClNO_4_^−^ | 341.0460 | −0.1 |
|  |  |  |  |  |  | 326.0221 | C_17_H_9_ClNO_4_^−^ | 326.0226 | −1.4 |
|  |  |  |  |  |  | 155.9846 | C_6_H_3_ClNO_2_^−^ | 155.9858 | −7.6 |
| a,b | 7.98 | 711.1307 | C_38_H_29_Cl_2_N_2_O_8_^−^ | 711.1306 | 0.1 | 696.1073 | C_37_H_26_Cl_2_N_2_O_8_^−^ | 696.1072 | 0.2 |
|  |  |  |  |  |  | 497.1029 | C_29_H_20_ClNO_5_^−^ | 497.1035 | −1.3 |
|  |  |  |  |  |  | 267.0761 | not identified | / | / |
|  |  |  |  |  |  | 155.9855 | C_6_H_3_ClNO_2_^−^ | 155.9858 | −1.8 |
|  |  |  |  |  |  | 34.9679 | Cl^−^ | 34.9694 | −42.9 |
| a | 8.32 + | 576.1548 | C_28_H_26_N_5_O_7_S^−^ | 576.1558 | −1.8 | 532.1153 | C_27_H_22_N_3_O_7_S^−^ | 532.1184 | −5.8 |
|  | 5.75 |  |  |  |  | 440.1097 | C_24_H_16_N_4_O_5_^−^ | 440.1126 | −6.6 |
|  |  |  |  |  |  | 396.0724 | not identified | / | / |
|  |  |  |  |  |  | 352.1058 | not identified | / | / |
|  |  |  |  |  |  | 306.0627 | C_17_H_10_N_2_O_4_^−^ | 306.0646 | −6.2 |
|  |  |  |  |  |  | 305.0530 | C_17_H_9_N_2_O_4_^−^ | 305.0568 | −12.4 |
|  |  |  |  |  |  | 289.0596 | C_17_H_9_N_2_O_3_^−^ | 289.0619 | −7.8 |
|  |  |  |  |  |  | 259.0610 | not identified | / | / |
|  |  |  |  |  |  | 212.0744 | C_10_H_14_NO_2_S^−^ | 212.0751 | −3.2 |
|  |  |  |  |  |  | 184.0391 | C_11_H_6_NO_2_^−^ | 184.0404 | −7.1 |
|  |  |  |  |  |  | 139.9923 | C_6_H_4_O_2_S^−^ | 139.9937 | −10.4 |
|  |  |  |  |  |  | 136.0425 | C_4_H_10_NO_2_S^−^ | 136.0438 | −9.4 |
| a,b | 9.15 + | 625.1527 | C_30_H_30_ClN_4_O_7_S^−^ | 625.1529 | −0.4 | 610.1273 | C_29_H_27_ClN_4_O_7_S^−^ | 610.1294 | −3.5 |
| **PR 5** | 6.5 |  |  |  |  | 355.0610 | C_19_H_14_ClNO_4_^−^ | 355.0617 | −1.9 |
|  |  |  |  |  |  | 352.1086 | not identified | / | / |
|  |  |  |  |  |  | 324.0432 | not identified | / | / |
|  |  |  |  |  |  | 212.0742 | C_10_H_14_NO_2_S^−^ | 212.0751 | −4.1 |
|  |  |  |  |  |  | 184.0402 | C_11_H_6_NO_2_^−^ | 184.0404 | −1.1 |
|  |  |  |  |  |  | 155.9842 | C_6_H_3_ClNO_2_^−^ | 155.9858 | −10.1 |
|  |  |  |  |  |  | 139.9926 | C_6_H_4_O_2_S^−^ | 139.9937 | −8.2 |
|  |  |  |  |  |  | 136.0422 | C_4_H_10_NO_2_S^−^ | 136.0438 | −11.6 |
| b | 9.67 | 439.1426 | C_25_H_19_N_4_O_4_^−^ | 439.1412 | 3.2 | 421.1267 | C_25_H_17_N_4_O_3_^−^ | 421.1306 | −9.3 |
|  |  |  |  |  |  | 306.0874 | C_17_H_12_N_3_O_3_^−^ | 306.0884 | −3.3 |
|  |  |  |  |  |  | 288.0776 | not identified | / | / |
|  |  |  |  |  |  | 168.0450 | C_11_H_6_NO^−^ | 168.0455 | −2.9 |
|  |  |  |  |  |  | 143.0522 | C_10_H_7_O^−^ | 143.0502 | 13.7 |
|  |  |  |  |  |  | 137.0354 | C_6_H_5_N_2_O_2_^−^ | 137.0357 | −1.8 |
|  |  |  |  |  |  | 107.0364 | C_6_H_5_NO^−^ | 107.0377 | −11.8 |
|  |  |  |  |  |  | 45.9936 | NO_2_^−^ | 45.9935 | 3.2 |

Table S2‑2 Detected features in LC-MS analysis of both Pigment Red 5 samples in the positive ion mode, which could be assigned to a plausible ion formula. The occurrence in the respective sample (a,b), their retention time (*t_R_*), and recorded mass of the precursor are listed. Ion formulae are proposed, including calculated *m*/*z* and mass deviation. Furthermore, recorded fragments including proposed ion formulae, calculated *m*/*z* and mass deviation are contained

| **Sample** | ***t_R_*** | **Precursor** | | | | **Fragments** | | | |
| --- | --- | --- | --- | --- | --- | --- | --- | --- | --- |
|  |  | **Recorded *m*/*z*** | **Ion formula** | **Calculated *m*/*z*** | **Δ *m*/*z* / ppm** | **Recorded *m*/*z*** | **Ion formula** | **Calculated *m*/*z*** | **Δ *m*/*z* / ppm** |
| a,b | 1.30 | 301.1218 | C_13_H_21_N_2_O_4_S^+^ | 301.1217 | 0.5 | 259.1115 | C_11_H_19_N_2_O_3_S^+^ | 259.1111 | 1.6 |
|  |  |  |  |  |  | 228.0317 | C_9_H_10_NO_4_S^+^ | 228.0325 | −3.5 |
|  |  |  |  |  |  | 186.0217 | C_7_H_8_NO_3_S^+^ | 186.0219 | −1.3 |
|  |  |  |  |  |  | 164.0703 | C_9_H_10_NO_2_^+^ | 164.0706 | −1.9 |
|  |  |  |  |  |  | 149.0465 | C_8_H_7_NO_2_^+^ | 149.0471 | −4.2 |
|  |  |  |  |  |  | 123.0456 | C_7_H_7_O_2_^+^ | 123.0441 | 12.5 |
|  |  |  |  |  |  | 95.0495 | C_6_H_7_O^+^ | 95.0491 | −3.8 |
|  |  |  |  |  |  | 72.0813 | C_4_H_10_N^+^ | 72.0808 | 7.3 |
| a,b | 1.54 | 458.1381 | C_22_H_24_N_3_O_6_S^+^ | 458.1380 | 0.1 | 440.1268 | C_22_H_22_N_3_O_5_S^+^ | 440.1275 | −1.5 |
|  |  |  |  |  |  | 304.0837 | C_18_H_12_N_2_O_3_^+^ | 304.0842 | −1.8 |
|  |  |  |  |  |  | 199.0495 | C_11_H_7_N_2_O_2_^+^ | 199.0502 | −3.5 |
|  |  |  |  |  |  | 169.0295 | not identified | / | / |
|  |  |  |  |  |  | 143.0501 | C_10_H_7_O^+^ | 143.0491 | 6.7 |
|  |  |  |  |  |  | 115.0539 | C_9_H_7_^+^ | 115.0542 | −2.8 |
|  |  |  |  |  |  | 72.0814 | C_4_H_10_N^+^ | 72.0808 | 8.7 |
| a,b | 2.25 | 244.1001 | C_11_H_18_NO_3_S^+^ | 244.1002 | −0.4 | 171.0107 | C_7_H_7_O_3_S^+^ | 171.0110 | −2.0 |
|  |  |  |  |  |  | 123.0434 | C_7_H_7_O_2_^+^ | 123.0441 | −5.3 |
|  |  |  |  |  |  | 107.0490 | C_7_H_7_O^+^ | 107.0491 | −1.3 |
|  |  |  |  |  |  | 92.0259 | C_6_H_4_O^+^ | 92.0257 | 2.5 |
|  |  |  |  |  |  | 77.0390 | C_6_H_5_^+^ | 77.0386 | 5.5 |
|  |  |  |  |  |  | 72.0809 | C_4_H_10_N^+^ | 72.0808 | 1.7 |
| a,b | 4.03 | 358.0836 | C_19_H_17_ClNO_4_^+^ | 358.0841 | −1.3 | 171.0432 | C_11_H_7_O_2_^+^ | 171.0441 | −5.0 |
|  |  |  |  |  |  | 143.0490 | C_10_H_7_O^+^ | 143.0491 | −1.0 |
|  |  |  |  |  |  | 115.0537 | C_9_H_7_^+^ | 115.0542 | −4.6 |
| a,b | 4.68 | 500.1885 | C_22_H_34_N_3_O_6_S_2_^+^ | 500.1884 | 0.3 | 363.1349 | C_18_H_23_N_2_O_4_S^+^ | 363.1373 | −6.6 |
|  |  |  |  |  |  | 256.1323 | not identified | / | / |
|  |  |  |  |  |  | 242.1170 | not identified | / | / |
|  |  |  |  |  |  | 228.1005 | C_14_H_14_NO_2_^+^ | 228.1019 | −6.2 |
|  |  |  |  |  |  | 212.0671 | C_13_H_10_NO_2_^+^ | 212.0706 | −16.5 |
|  |  |  |  |  |  | 182.0592 | not identified | / | / |
|  |  |  |  |  |  | 72.0803 | C_4_H_10_N^+^ | 72.0808 | −6.6 |
| a,b | 6.59 | 308.1026 | C_17_H_14_N_3_O_3_^+^ | 308.1030 | −1.2 | 291.0996 | C_17_H_13_N_3_O_2_^+^ | 291.1002 | −2.2 |
|  |  |  |  |  |  | 274.0938 | not identified | / | / |
|  |  |  |  |  |  | 156.0443 | C_10_H_6_NO^+^ | 156.0444 | −0.6 |
|  |  |  |  |  |  | 152.0580 | C_7_H_8_N_2_O_2_^+^ | 152.0580 | −0.2 |
|  |  |  |  |  |  | 128.0494 | C_9_H_6_N^+^ | 128.0495 | −0.6 |
|  |  |  |  |  |  | 106.0648 | C_7_H_8_N^+^ | 106.0651 | −3.1 |
|  |  |  |  |  |  | 79.0535 | C_6_H_7_^+^ | 79.0542 | −9.2 |
|  |  |  |  |  |  | 77.0393 | C_6_H_5_^+^ | 77.0386 | 9.4 |
| Continued on next page | | | | | | | | | |
| Table S2-2 - continued from previous page | | | | | | | | | |
| **Sample** | **t_R_** | **Precursor** | | | | **Fragments** | | | |
|  |  | **Recorded *m*/*z*** | **Ion formula** | **Calculated *m*/*z*** | **Δ *m*/*z* / ppm** | **Recorded *m*/*z*** | **Ion formula** | **Calculated *m*/*z*** | **Δ *m*/*z* / ppm** |
| a,b | 6.69 | 727.1602 | C_39_H_33_Cl_2_N_2_O_8_^+^ | 727.1608 | −0.9 | 370.0835 | C_20_H_17_ClNO_4_^+^ | 370.0841 | −1.5 |
|  |  |  |  |  |  | 352.0700 | C_20_H_15_ClNO_3_^+^ | 352.0735 | −9.9 |
|  |  |  |  |  |  | 183.0436 | C_12_H_7_O_2_^+^ | 183.0441 | −2.5 |
| a,b | 8.06 | 713.1438 | C_38_H_31_Cl_2_N_2_O_8_^+^ | 713.1452 | −2.0 | 526.1054 | C_30_H_21_ClNO_6_^+^ | 526.1052 | 0.4 |
|  |  |  |  |  |  | 339.0646 | C_19_H_14_ClNO_3_^+^ | 339.0657 | −3.2 |
|  |  |  |  |  |  | 283.0752 | not identified | / | / |
|  |  |  |  |  |  | 255.0785 | not identified | / | / |
| a | 8.32 + | 578.1701 | C_28_H_28_N_5_O_7_S^+^ | 578.1704 | −0.5 | 440.1274 | C_22_H_22_N_3_O_5_S^+^ | 440.1275 | −0.2 |
|  | 5.75 |  |  |  |  | 304.0837 | C_18_H_12_N_2_O_3_^+^ | 304.0842 | −1.8 |
|  |  |  |  |  |  | 199.0504 | C_11_H_7_N_2_O_2_^+^ | 199.0502 | 1.0 |
|  |  |  |  |  |  | 169.0296 | not identified | / | / |
|  |  |  |  |  |  | 143.0481 | C_10_H_7_O^+^ | 143.0491 | −7.3 |
|  |  |  |  |  |  | 72.0814 | C_4_H_10_N^+^ | 72.0808 | 8.7 |
| a,b | 9.15 + | 627.1671 | C_30_H_32_ClN_4_O_7_S^+^ | 627.1675 | −0.6 | 440.1277 | C_22_H_22_N_3_O_5_S^+^ | 440.1275 | 0.5 |
| **PR 5** | 6.55 |  |  |  |  | 305.0903 | C_18_H_13_N_2_O_3_^+^ | 305.0921 | −5.8 |
|  |  |  |  |  |  | 199.0500 | C_11_H_7_N_2_O_2_^+^ | 199.0502 | −1.0 |
|  |  |  |  |  |  | 169.0296 | not identified | / | / |
|  |  |  |  |  |  | 143.0481 | C_10_H_7_O^+^ | 143.0491 | −7.3 |
|  |  |  |  |  |  | 72.0811 | C_4_H_10_N^+^ | 72.0808 | 4.5 |

# LC-EC-MS

## Electrochemical transformation of PR 5

Fig. S3‑1 MS/MS spectrum of oxidative transformation product (OTP) OTP(+)358 in the positive ion mode obtained by LC-EC-MS/MS analysis. The TP is named by the electrochemical conditions (oxidative = OTP), the polarity (+) in which it was observed, and the respective nominal *m*/*z*

## Electrochemical transformation of selected byproducts

### Impurity C_28_H_27_N_5_O_7_S, PR 5a

The impurity profiling revealed the presence of a second NAS pigment in sample PR 5a. To compare the electrochemical transformation processes occurring in NAS structures, the TPs of this impurity are characterized in both polarities and oxidative and reductive conditions. Fig. S3‑2 shows the EICs of *m*/*z* 576.156 and its OTPs in the negative ion mode and potentials from 0.0 to 2.0 V.


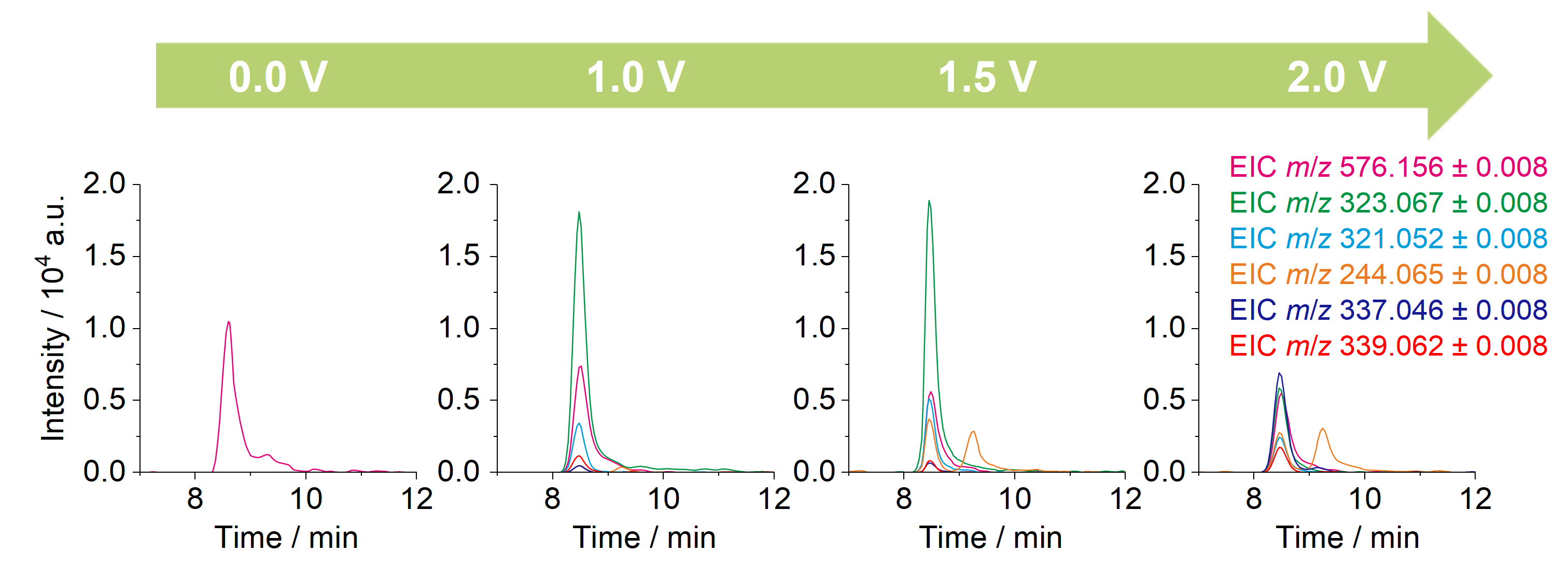


Fig. S3‑2 Extracted ion chromatograms (EICs) of *m*/*z* 576.156 and its oxidative transformation products observed at the same retention time, obtained by online-LC-EC-MS in the negative ion mode. Oxidative runs at 0.0 V, 1.0 V, 1.5 V, and 2.0 V are shown for the sample PR 5a

Table S3‑1 lists the observed TPs of this impurity in oxidative and reductive conditions in both polarities. Ion formulae are proposed, including calculated *m*/*z* and mass deviation. The structural resemblance of PR 5 and the impurity become apparent in the TPs, that form at both their retention times (OTP(−)244, OTP(+)244, and RTP(+)259).

Table S3‑1 List of transformation products (TPs) of impurity C_28_H_27_N_5_O_7_S, detected in the negative and positive ion modes and assigned to their corresponding parent compound by retention time filtering. TPs were detected in sample PR 5a. The potential with highest intensity of the TP is listed and ion formulae, including calculated *m*/*z* and mass deviation, are proposed. TPs are named by the electrochemical conditions (oxidative = OTP, reductive = RTP), the polarity (+/−) in which they were observed, and their respective nominal *m*/*z*

| **Potential / V** | **Recorded *m*/*z*** | **Ion formula** | **Calculated *m*/*z*** | **Δ *m*/*z* / ppm** | **Name** |
| --- | --- | --- | --- | --- | --- |
| **Negative ion mode** | | | | | |
| +2.0 (Ox) | 339.0622 | C_17_H_11_N_2_O_6_^−^ | 339.0623 | −0.2 | OTP(−)339 |
| +2.0 (Ox) | 337.0465 | C_17_H_9_N_2_O_6_^−^ | 337.0466 | −0.3 | OTP(−)337 |
| +1.0 (Ox) | 323.0679 | C_17_H_11_N_2_O_5_^−^ | 323.0673 | 1.7 | OTP(−)323 |
| +1.5 (Ox) | 321.0518 | C_17_H_9_N_2_O_5_^−^ | 321.0517 | 0.3 | OTP(−)321 |
| +1.5 (Ox) | 244.0648 | C_10_H_14_NO_4_S^−^ | 244.0649 | −0.4 | OTP(−)244 |
| −2.0 (Red) | 322.0844 | C_17_H_12_N_3_O_4_^−^ | 322.0833 | 3.3 | RTP(−)322 |
| −2.0 (Red) | 306.0894 | C_17_H_12_N_3_O_3_^−^ | 306.0884 | 3.2 | RTP(−)306 |
| **Positive ion mode** | | | | | |
| +2.0 (Ox) | 244.1008 | C_11_H_18_NO_3_S^+^ | 244.1002 | 2.5 | OTP(+)244 |
| −2.0 (Red) | 259.1118 | C_11_H_19_N_2_O_3_S^+^ | 259.1110 | 3.1 | RTP(+)259 |

For structural annotation, the MS/MS spectra were evaluated. The spectra including suggested structures are depicted in Fig. S3‑3. For RTP(−)322, no fragment spectrum could be obtained. Furthermore, for OTP(+)339, no clean MS/MS spectrum could be acquired, as there was a close *m*/*z* in the isolation width of the quadrupole (= 1.3 *m*/*z*). The corresponding MS^1^ spectra at 1.0 V and 2.0 V are depicted in Fig. S3‑4.


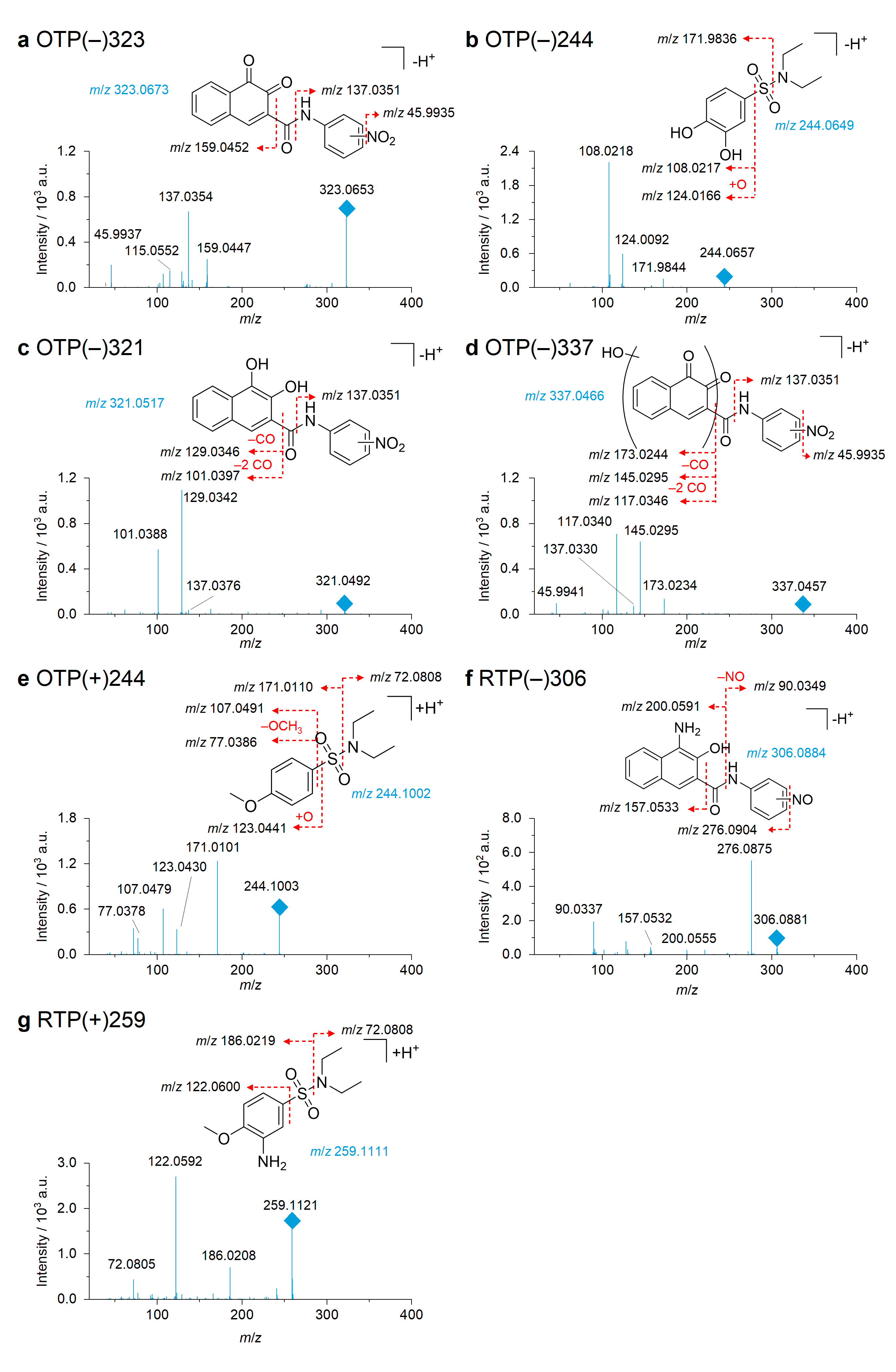


Fig. S3‑3 MS/MS spectra of oxidative (OTP) and reductive (RTP) transformation products of C_28_H_27_N_5_O_7_S in the negative and the positive ion mode, respectively, obtained by online-LC-EC-MS/MS analysis. TPs are named by the electrochemical conditions (oxidative = OTP, reductive = RTP), the polarity (+/−) in which they were observed, and their respective nominal *m*/*z*


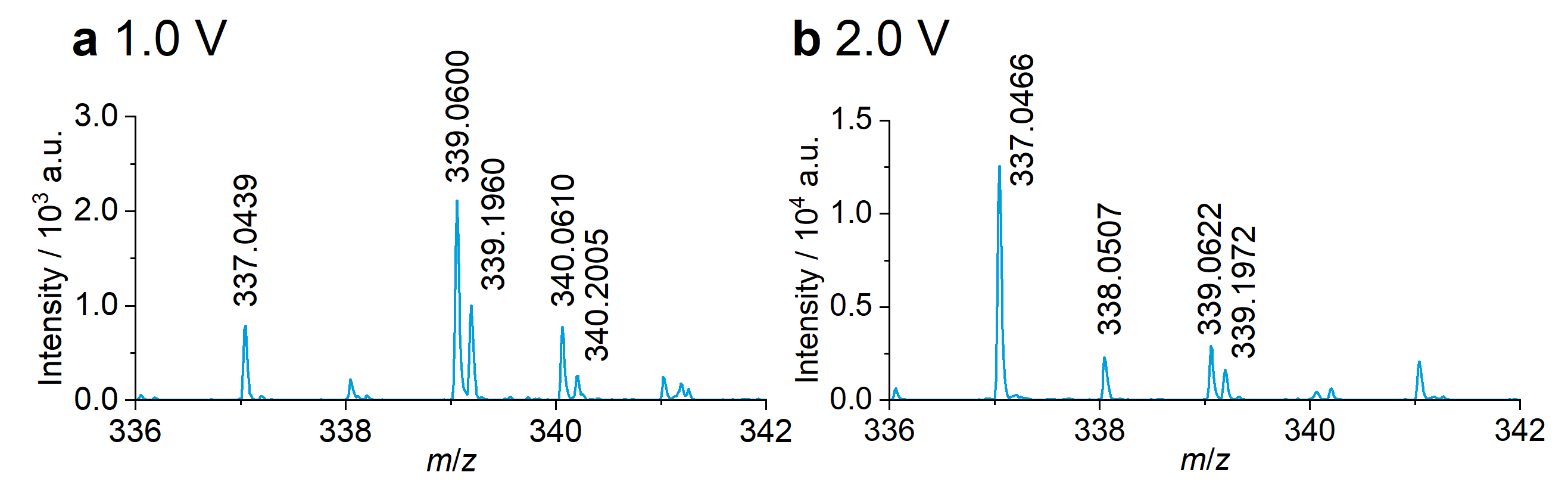


Fig. S3‑4 Zoom of averaged MS^1^ spectra at *t_R_*= 8.41 to 8.45 min of PR 5a obtained by online-LC-EC-MS in the negative ion mode. Spectra were extracted from oxidative runs at 1.0 V (a) and 2.0 V (b)

The proposed structures and derived electrochemical transformation pathways of impurity C_28_H_27_N_5_O_7_S are depicted in Fig. S3‑5 for oxidation and Fig. S3‑6 for reduction. The oxidative transformation route is comparable to the one observed for PR 5 and discussed in the main document. The oxidative azo cleavage leads to formation of phenolic hydroxylated compounds. A further oxidation of OTP(−)323 yields an *ortho*-quinone through subsequent dehydrogenation. Additionally, OTP(−)339 and OTP(−)337 are both further hydroxylated at the naphthol ring.


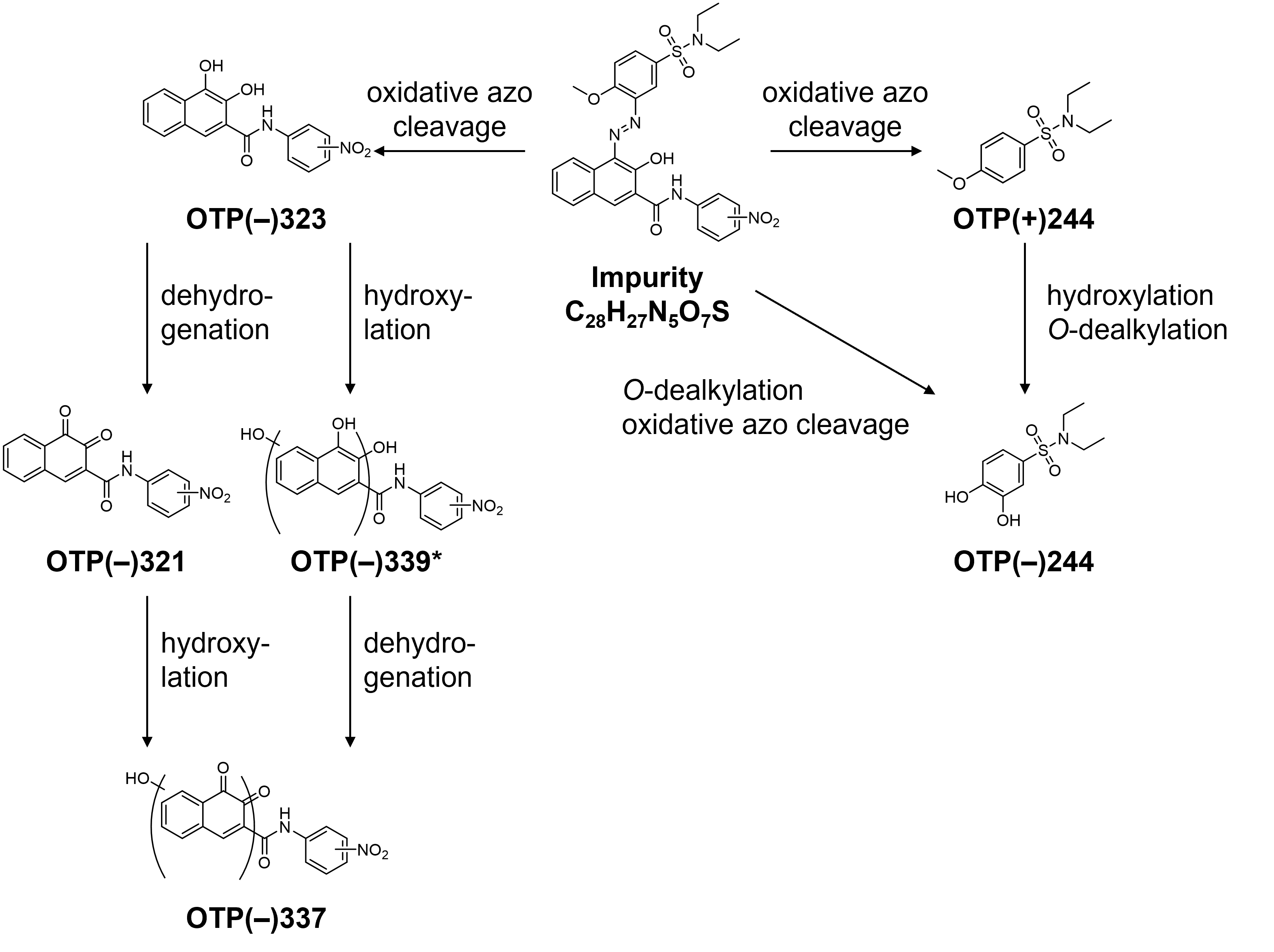


Fig. S3‑5 Oxidative electrochemical transformation routes of impurity C_28_H_27_N_5_O_7_S observed by online-LC-EC-MS. Oxidative transformation products (OTPs) are named by their observed polarity (+/−) and their respective nominal *m*/*z*. The position of the NO_2_ substitution, and the positions of hydroxylation for OTP(−)339 and OTP(−)337 cannot be determined unequivocally based on the acquired data. Hydroxylation after oxidative cleavage is displayed according to the most likely position for hydroxylation (OTP(−)323 and OTP(−)244). *For OTP(−)339 no clean MS/MS could be obtained due to *m*/*z* overlap in the isolation width of the quadrupole

Under reductive electrochemical conditions, primary aromatic amines (PAAs) are formed by reductive azo cleavage (RTP(−)322 and RTP(+)259). Here, a further reaction step reduces the nitro-group to a nitroso group, forming RTP(−)306.


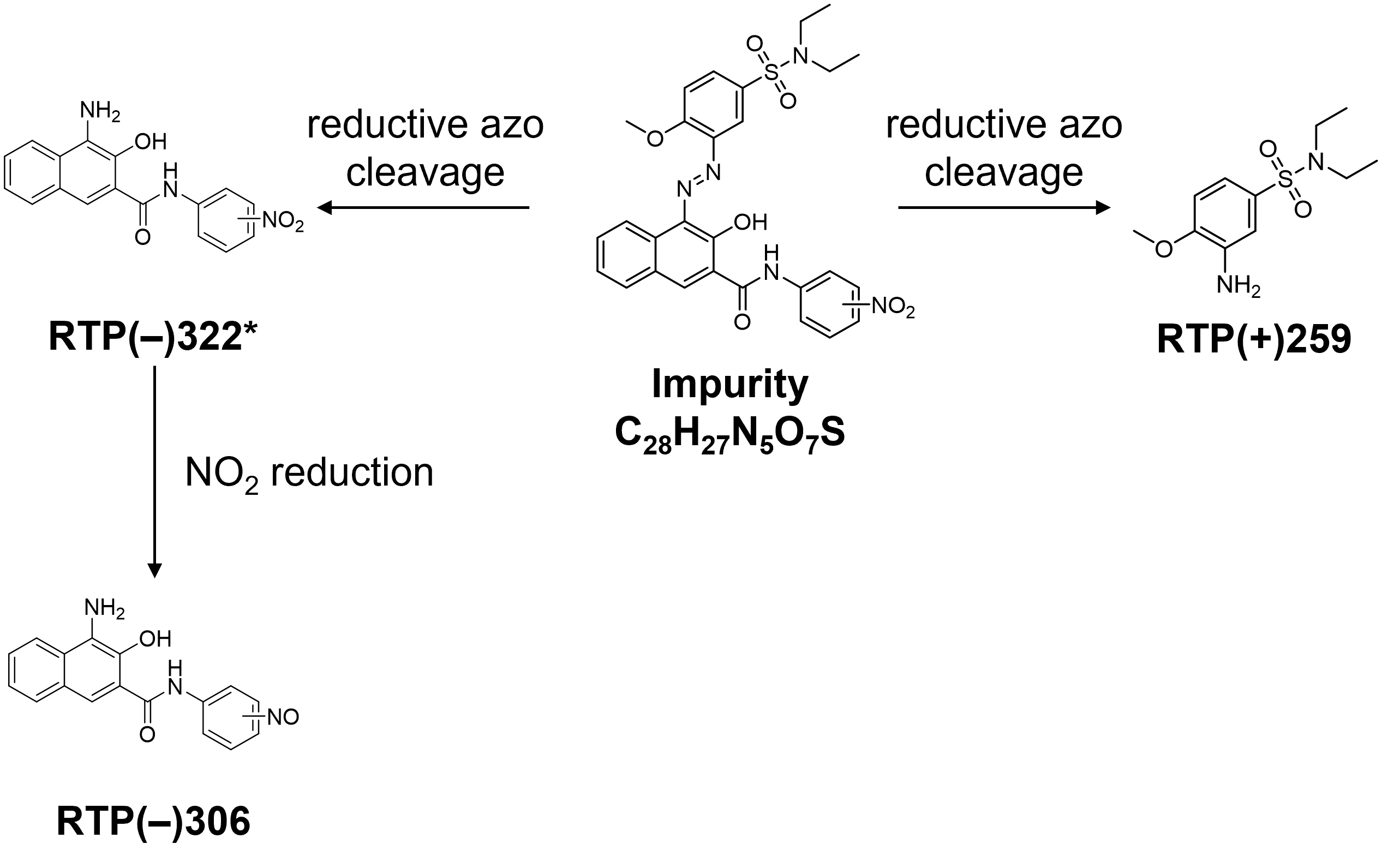


Fig. S3‑6 Reductive electrochemical transformation routes of impurity C_28_H_27_N_5_O_7_S observed by online-LC-EC-MS. Reductive transformation products (RTPs) are named by their observed polarity (+/−) and their respective nominal *m*/*z*. The position of the NO_2_ substitution cannot be determined unequivocally based on the acquired data. *For RTP(−)322 no MS/MS was obtained

### Impurity C_19_H_16_ClNO_4_, PR 5a and PR 5b

Azo pigments are produced by azo coupling. The coupling component for NAS pigments is modified NAS and the NAS derivative for PR 5 synthesis was detected in both samples. As this might be a common impurity, the TPs for this compound were also evaluated.

Table S3‑2 lists the *m*/*z* of the observed TP in oxidative and reductive conditions. For the one observed TP, an ion formula is proposed, including calculated *m*/*z* and mass deviation. This corresponds to one hydroxylation of the structure and results in the same *m*/*z* as observed by the oxidative azo cleavage of PR 5 at 1.0 V. Fig. S3‑7 shows the EICs over the whole chromatographic run at 1.0 V exemplary for sample PR 5a in the negative ion mode.

Table S3‑2 List of transformation products (TPs) of impurity C_19_H_16_ClNO_4_ detected in negative and the positive ion mode assigned to their corresponding parent compound by retention time filtering. The TP was detected in both PR 5 samples. The potential with highest intensity of the TP is listed and ion formulae, including calculated *m*/*z* and mass deviation, are proposed. The TP is named by the electrochemical conditions (oxidative = OTP), the polarity (−) in which it was observed, and the respective nominal *m*/*z*

| **Potential / V** | **Recorded *m*/*z*** | **Ion formula** | **Calculated *m*/*z*** | | **Δ *m*/*z* / ppm** | **Name** |
| --- | --- | --- | --- | --- | --- | --- |
| **Negative ion mode** | | | | | | |
| +1.0 (Ox) | 372.0647 | C_19_H_15_ClNO_5_^−^ | | 372.0644 | 0.7 | OTP(−)372 |
| **Positive ion mode** | | | | | | |
| - | | | | | | |


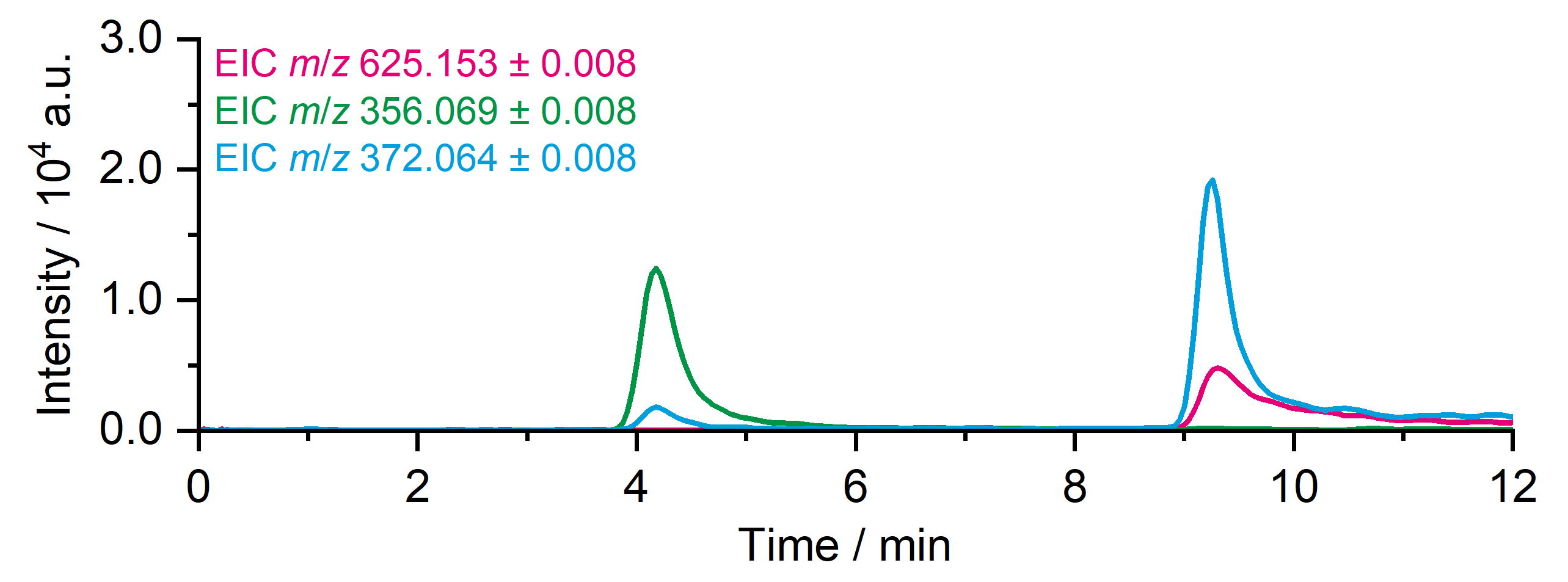


Fig. S3‑7 Extracted ion chromatograms (EICs) of PR 5 (*m*/*z* 625.153), its synthesis precursor (*m*/*z* 356.069) and their common oxidative transformation product observed at the same retention time, obtained by online-LC-EC-MS in the negative ion mode. Depicted is the run at 1.0 V of PR 5a

Fig. S3‑8 shows the oxidation route of the synthesis precursor. According to the proposed ion formula, the detected OTP corresponds to a single hydroxylation. Due to the lack of MS/MS data, the position of the hydroxylation of the impurity cannot be determined.


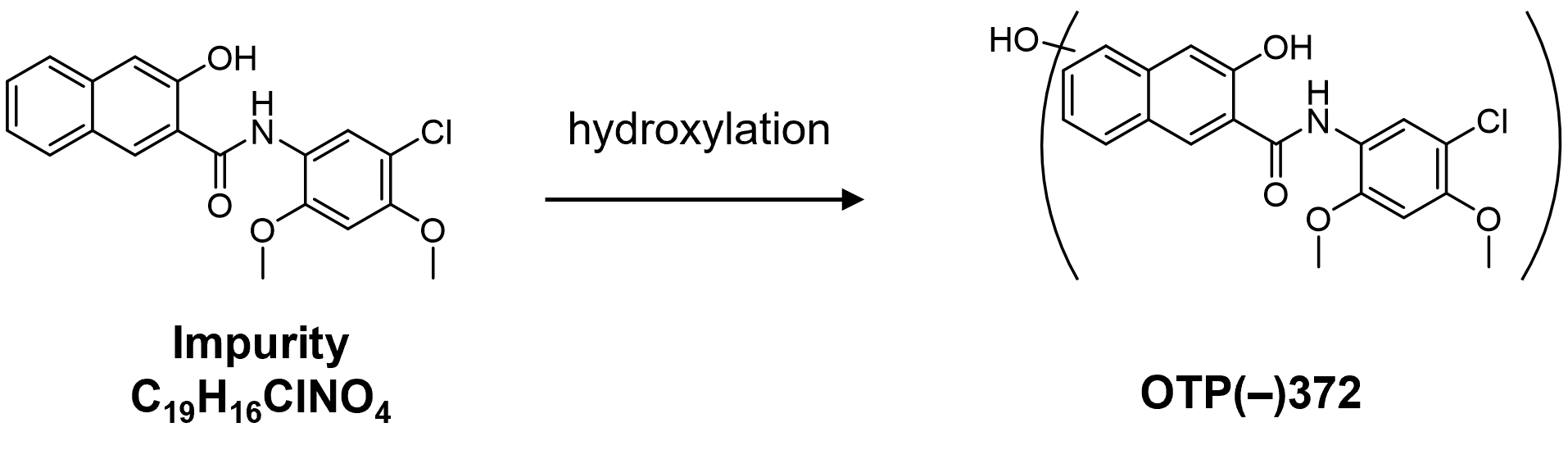


Fig. S3‑8 Oxidative electrochemical transformation route of modified naphthol AS, impurity C_19_H_16_ClNO_4_, observed by online-LC-EC-MS. The oxidative transformation product (OTP) is named by the observed polarity (−) and the respective nominal *m*/*z*. The position of the hydroxylation cannot be determined unequivocally based on the acquired data

### Impurity C_17_H_12_N_2_O_4_, PR 5a

The corresponding nitro-substituted NAS coupling component for the second NAS pigment in sample PR 5a was also investigated for occurring TPs. Table S3‑3 lists the *m*/*z* the observed TPs in oxidative and reductive conditions. Ion formulae are proposed, including calculated *m*/*z* and mass deviations. Similar to the coupling component for PR 5, only one TP could be identified. Under reductive conditions, the nitro group is reduced, comparable to the transformation pathway for impurity C_28_H_27_N_5_O_7_S. Fig. S3‑9 shows the EICs over the whole chromatographic run at −2.0 V for sample PR 5a in the negative ion mode. *m*/*z* 307.072 also shows a peak at the retention time of the second NAS pigment. This corresponds to the isotope pattern of its RTP(−)306. In positive ion mode, the parent compound is not detectable. Filtering for products in the corresponding retention time window in positive ion mode still did not reveal the presence of any additional TPs.

Table S3‑3 List of transformation products (TPs) of impurity C_17_H_12_N_2_O_4_ detected in the negative and the positive ion modes assigned to their corresponding parent compound by retention time filtering. The TP was detected in sample PR 5a. The potential with highest intensity of the TP is listed and ion formulae, including calculated *m*/*z* and mass deviation, are proposed. The TP is named by the electrochemical conditions (reductive = RTP), the polarity (−) in which it was observed, and the respective nominal *m*/*z*

| **Potential / V** | **Recorded *m*/*z*** | **Ion formula** | **Calculated *m*/*z*** | | **Δ *m*/*z* / ppm** | **Name** |
| --- | --- | --- | --- | --- | --- | --- |
| **Negative ion mode** | | | | | | |
| −2.0 (Red) | 291.0781 | C_17_H_11_N_2_O_3_^−^ | | 291.0775 | 2.0 | RTP(−)291 |
| **Positive ion mode** | | | | | | |
| - | | | | | | |


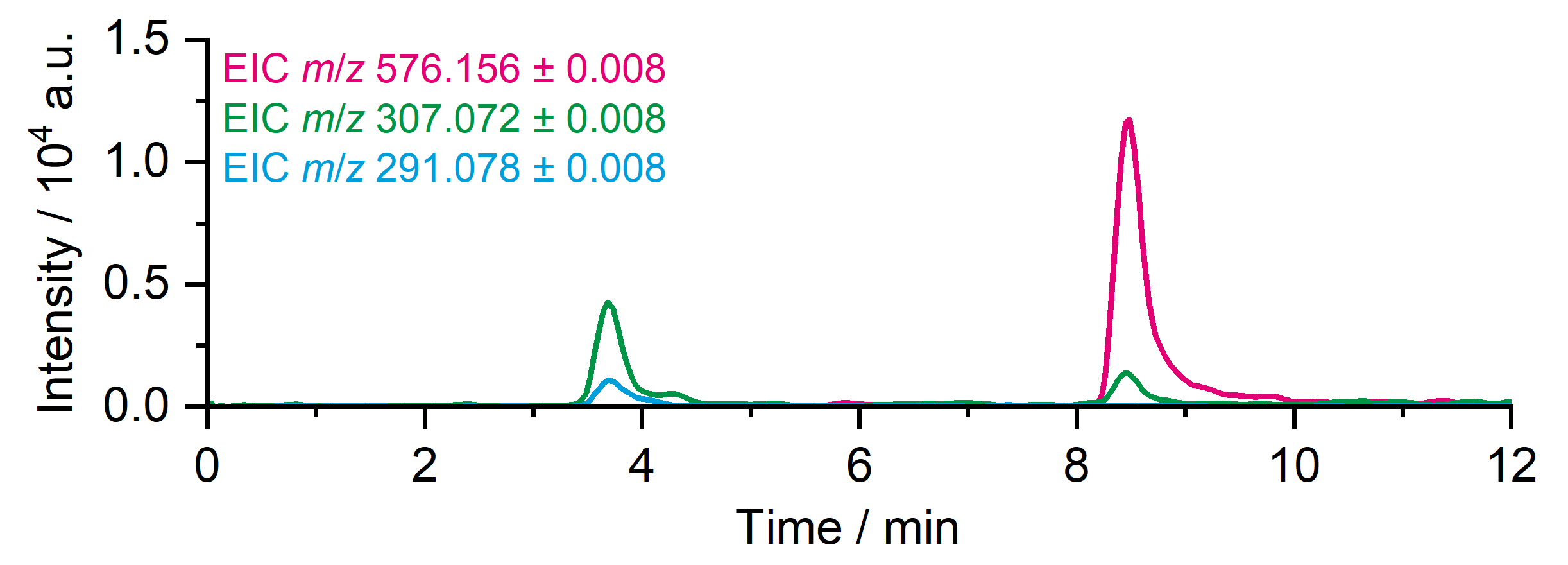


Fig. S3‑9 Extracted ion chromatograms (EICs) of the second NAS pigment (*m*/*z* 576.156), its synthesis precursor (*m*/*z* 307.072) and a reductive transformation product of *m*/*z* 307.072 observed at the same retention time, obtained by online-LC-EC-MS in the negative ion mode and at −2.0 V

Fig. S3‑10 shows the corresponding electrochemical reduction route. The position of the NO_2_ and NO substitution cannot be determined unequivocally based on the acquired data


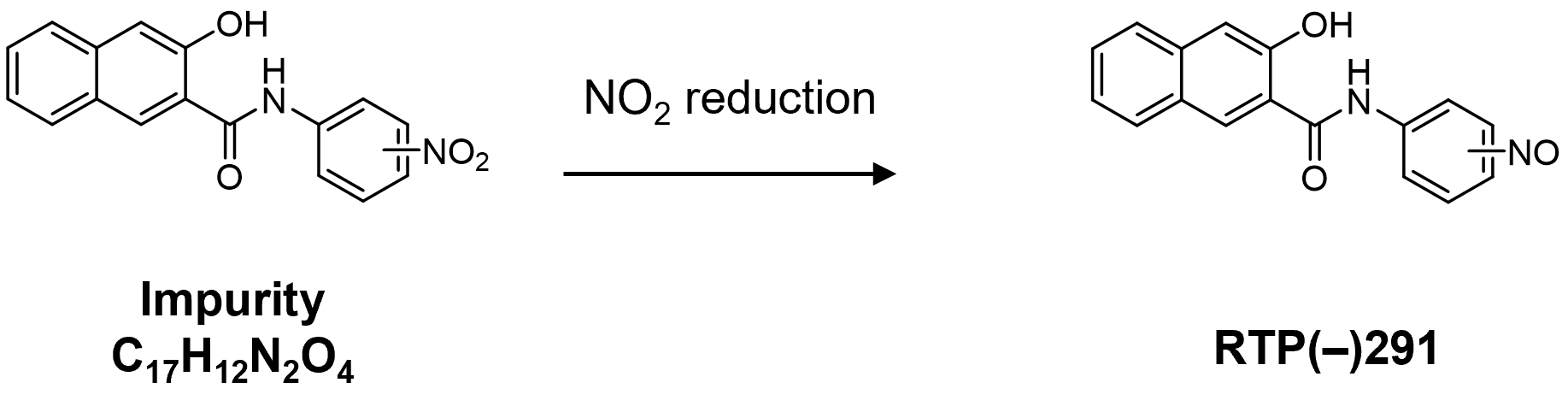


Fig. S3‑10 Reductive electrochemical transformation route of modified naphthol AS, impurity C_17_H_12_N_2_O_4_, observed by online-LC-EC-MS. The reductive transformation product (RTP) is named by the observed polarity (−) and the respective nominal *m*/*z*. The position of the NO_2_ and NO substitution cannot be determined unequivocally based on the acquired data
